# The disease burden of gout in Asian countries and regions from 1990 to 2021, risk factors and forecast analysis: A systematic study of Asian disease burden in 2021

**DOI:** 10.1371/journal.pone.0328543

**Published:** 2025-07-28

**Authors:** Fuyu Yang, Runze Chen, Jun Xiong, Wenzhen Wang, Peng Yu, Huilin Wang

**Affiliations:** 1 School of Public Administration, Hangzhou Normal University, Hangzhou, Zhejiang, China; 2 Engineering Research Center of Mobile Health Management System, Ministry of Education, Hangzhou, China; Guru Nanak College, INDIA

## Abstract

**Objective:**

Use the Global Burden of Disease (GBD) database to obtain gout’s disease burden indicators, risk factors, 1990–2021 data in Asian countries/regions and 2050 forecast data for gout prevention and control basis.

**Methods:**

Collect gout prevalence, incidence etc. from global burden of disease data in Asia, stratified by gender and age, analyze with statistical charts. Use Joinpoint regression model for trend analysis. Predict 2050 East Asia incidence rate with Bayesian Age-Period-Cohort (BAPC) model.

**Results:**

1990 - 2021, Age-standardized Prevalence Rate (ASPR) and Age-standardized Incidence Rate (ASIR) rose globally, in Asia and SDI regions. East Asia’s incidence rose fast. Men’s ASPR is nearly three times that of women. 55–59 – year – olds have the highest incidence rate in 2021. High BMI patients are common, and low proportion of kidney dysfunction impairment. East Asia’s ASIR will rise by 2050 but slower.

**Conclusion:**

Gout’s disease burden rose in Asia 1990–2021. Prevalence and incidence vary by country/region, especially in East Asia. Men’s prevalence is higher. 55–59 – year – olds have the highest prevalence. In Asia’s developed countries, prevalence and incidence are above the global average in 2021. High BMI and kidney dysfunction impairment are key risk factors. These findings necessitate preventive strategies, enhanced cooperation in prevention and control management, public awareness campaigns, and early treatment interventions.

## Introduction

Gout is a chronic non-communicable disease and a treatable form of arthritis, which is caused by an increase in uric acid concentration to a level that precipitates monosodium urate crystals (MSU) is considered to be the most important biochemical basis for gout [1]. The cause of gout may be that the patient eats a large amount of purine-rich foods or produces too much uric acid due to certain diseases, which exceeds the kidneys clearance capacity, causing an increase in blood uric acid, causing urate crystals to be deposited in body fluids and other tissues. Within the body, it is phagocytosed by white blood cells to produce inflammation, and repeated attacks can lead to gout [2]. At the same time, gout is closely related to metabolic problems, and high BMI index and impaired kidney dysfunction are considered risk factors for gout [3,4]. The main symptoms of gout include red, swollen, hot and painful joints, yellow urine, dry mouth and bitter mouth, fatigue, chest and epigastrium tightness, pain, limited movement of joints, swelling and deformation of joints, etc. These symptoms generally reduce the quality of life of patients, and the vast majority of patients will have recurring attacks. Gout patients usually endure joint pain, deformity, disability, huge financial burden, psychological pressure and the adverse reactions of long-term use of analgesics, especially It is caused by recurrent attacks and complications [5]. This significantly impairs patients’ quality of life. Compounding this burden, studies show unsatisfactory treatment compliance among gout patients. Poor treatment compliance not only has a negative impact on the patient s life and personal health, but also brings hidden dangers to the medical system. Furthermore, the medical expenditures and workplace productivity losses associated with gout have created dual economic burdens, compounding regional socioeconomic pressures through both direct healthcare costs and indirect workforce impacts [6]. There are a large number of gout patients in Asia, and in a gender survey of patients, men are significantly more common than women. The prevalence is higher in the middle-aged and elderly groups. And with changes in lifestyle and the aging of the population, the prevalence is on the rise. From previous studies on gout, it has been shown that the prevalence of gout has continued to increase worldwide in the past and is likely to increase further in the coming years due to changes in dietary intake and increased lifespan [7,8]. With the increasing incidence of gout, research on this condition across different regions has been continuously advancing. In the realm of gout epidemiological studies, previous investigations have illuminated the regional characteristics of gout burden in Asia from various perspectives. Data derived from multicenter surveys in China indicate that the prevalence of gout stands at 1.1%, with rates of 1.9% in males and 0.3% in females, demonstrating significant spatial differentiation characterized by higher prevalence in coastal areas compared to inland regions. This phenomenon is notably correlated with the frequency of seafood consumption and the level of urbanization [1]. An analysis based on the Global Burden of Disease (GBD) 2017 data further corroborates that from 1990 to 2017, the disability-adjusted life year (DALY) rate for gout in China surged by 58.6%, with individuals aged 45–64 accounting for 62% of this increase, thereby underscoring the amplifying effect of population aging on the disease burden [9]. Regarding risk factor research, studies have revealed that the prevalence of hyperuricemia in China escalated from 1.4% in 1980 to 8.4% in 2008, closely associated with the Westernization of dietary patterns accompanying economic development [2] Furthermore, research that incorporates the perspective of chronic kidney disease highlights that the synergistic effect of hyperuricemia and renal insufficiency can elevate the risk of cardiovascular events in gout patients by 3.2 times [4].

Although numerous analyses have examined the global burden of gout disease, most are conducted at a global or national level and lack a detailed analysis of the heterogeneity in disease burden across sub-regions in Asia. This limitation makes it challenging to identify differences in the rate of incidence increase and exposure patterns to risk factors across regions with varying Socio-demographic Index (SDI). Additionally, there is insufficient quantification of risk factors specific to the Asian population, and the attributable differences in metabolic factors, such as high BMI and Kidney dysfunction, across regions remain unclear. Globally, regional variations in the incidence of gout exist. Investigating the incidence of gout in Asia will help understand the actual status of gout in the region and take targeted measures based on this. Among all regions in Asia, the disease burden of gout is particularly prominent in Southeast Asia. Therefore, prediction and analysis of gout incidence in East Asia is of great significance for formulating effective public health policies and interventions. This article will analyze the disease burden of gout in Asia based on GBD research data, focusing on Asia. It aims to describe the disease burden of gout in Asia from 1990 to 2021 by gender, age, regional distribution, etc. Predict the development trend of gout in 2050 and provide data support for formulating strategies and measures to prevent and control gout disease. The study establishes a refined analytical framework for the burden of gout in Asia by integrating the latest GBD2021 dataset, thereby overcoming the limitations of previous global studies that conducted macro-level analyses based on national units. At the level of risk factor research, it systematically quantifies the regional attributable risks of high BMI and Kidney dysfunction for the Asian population with specific genetic backgrounds, addressing the gap in research on metabolic risk factors unique to Asia. Additionally, the study employs the Bayesian Age-Period-Cohort (BAPC) model to predict the development trends of gout in East Asia by 2050, thereby supplementing the predictions of traditional models.

## Materials and methods

### Overview

This study uses data extracted from the GBD, which provides a complete analysis of all available data from 1990 to 2021, thereby superseding all previously published GBD study estimates. All GBD 2021 estimates are available via the following link: (https://vizhub.healthdata.org/gbd-results/). Joinpoint regression analysis model was used to analyze the characteristics and trends of gout Age-standardized Incidence Rate (ASIR), Age-standardized Prevalence Rate (ASPR) and DALYs in Asia from 1990 to 2021. Finally, BAPC was used to predict the incidence of gout in East Asia in 2050. In this study, the R software package (version 4.2.3) and JD _ GBDR (V2.32, Jingding Medical Technology Co., Ltd.) was used for the drawing of the ﬁgures. The specific workflow was as follows: Gout burden data for Asia from 1990 to 2021, extracted from the GBD 2021 database, were uploaded and loaded into the system. The data were then meticulously filtered by configuring parameters including time range, regional classification, age stratification, and sex groups. Subsequently, adjusted models were employed for statistical analysis and chart generation. As this study uses publicly available data from the GBD 2021 database, which has been de-identified and does not involve direct individual interventions or the collection of personal privacy information, the ethics committee has approved the waiver of informed consent.

Because gout is non-fatal, it is not directly related to mortality. Therefore, there are no years of life lost (YLL). Therefore, this study uses the prevalence, incidence, age-standardized rates, and 95% uncertainty interval (95% UI) of Asian countries and regions from 1990 to 2021, and studies separately by gender, region, and risk factors. And incorporate primary data sources including prevalence and incidence into the estimation process. However, studies with the following characteristics were excluded: (1) subgroups not representative of the entire population; (2) not population-based studies; (3) small sample size (less than 150); (4) retrospective studies, not original research [9].

### Case definitions and data sources

We used the International Classification of Diseases, 10th Revision, to classify and code diseases. The disease classification code for gout is M10 [10]. And the standards established by the American College of Rheumatology in 1977 were adopted [11]. The criteria include the presence of MSU crystals in synovial fluid or the presence of tophus proven to contain MSU crystals and at least 6 of 12 symptoms of gout, or findings including the following the patient presents with an acute arthritis attack, with inflammation peaking within a day, a single Arthritis attack, observed joint erythema, first MTP joint pain or swelling, unilateral attack involving the first MTP joint, unilateral attack involving the tarsus, suspected medial phlegm, hyperuricemia, intra-articular insufficiency on X-ray Symmetrical swelling and negative culture of synovial fluid microorganisms during an episode of joint inflammation make the diagnosis [12]. Prevalence Incidence and DALYs: Prevalence is the proportion of people in a population who have a disease, injury, or sequelae. Incidence is the number of new cases caused by a specific cause in a given population during a given period. Use similar principles to identify, enhance comparability and analyze data to estimate prevalence、incidence and DALY. DALY is a comprehensive indicator that quantitatively calculates the loss of healthy life years due to premature death and disability caused by various diseases. It is measured by combining losses due to premature death (the difference between actual years of death and life expectancy at that age for people with low mortality) and health losses due to disability. Disability-adjusted life years can scientifically indicate serious health-threatening diseases and major health problems in the region.

### Uncertainty analysis

In the GBD study, each estimate was calculated 1,000 times, each time sampling from the distribution, rather than sampling point estimates from the data input, data transformation model selections. The 95th UI is determined by the 25th and 975th values of 1000 values, and then sorted from small to large. These UIs include uncertainties stemming from data sample size, adjustments for different sources of all-cause mortality, and specification and estimation of cause-of-death models [9].

### Risk factors

We extracted attributable risks from GBD for two metabolic risk factors: high body mass index (BMI) and kidney dysfunction. BMI is the metric currently used to define and classify adult anthropometric height/weight characteristics. The unit is kg/m2. BMI. We also analyzed obesity and kidney dysfunction as risk factors for gout, and studied the impact of each risk factor on gout DALYs in different age groups.

### Statistical analysis

**Prevalence** and incidence are fundamental indicators of disease trends. We conducted an analysis of the incidence and prevalence of gout across various Asian countries and regions, categorized by age, gender, year, and region. The incidence rate was calculated as the ratio of new gout cases in the target Asian population in 2021 to the exposed population during the same period, expressed as the number of new cases per 100,000 individuals. The prevalence was determined as the ratio of the total number of new and existing gout cases in the target Asian population in 2021 to the average population, measured as cases per 100,000 individuals. To ensure comparability of statistical data, we employed age-standardized incidence rates to assess the disease burden of gout. Metrics such as the ASIR and the Age-Standardized DALY rate account for variations in the population’s age composition. Age standardization aims to mitigate the impact of the population’s age structure, thereby enhancing the comparability of study indicators. In the GBD database, these metrics are estimated using the world population age standard, calculated as follows: ASR = $\frac{\sum_{i=1}^{A}\;a_{i}w_{i}}{\sum_{i=1}^{A}\;w_{i}}$ ×100,000, it is the sum of the age-specific rates (ai, where i represents the ith age group) multiplied by the number of individuals in the corresponding age subgroup (wi), divided by the sum of the standard population. And perform descriptive analysis and visual display of gout-related epidemiological data according to gender, age, region and year. This study employed Joinpoint regression modeling to analyze the spatiotemporal trends of ASIR, mortality, and DALYs for gout in Asia from 1990 to 2021. The method identifies inflection points (joinpoints) within time-series data, partitioning it into consecutive segments where log-linear models quantify trend variations. Specifically, potential joinpoint locations were systematically evaluated using a grid search algorithm, with the optimal number determined through permutation testing. A maximum of four joinpoints were permitted to prevent model overfitting. Annual percentage change (APC) for each segment was calculated with 95% confidence intervals derived from Monte Carlo simulations, and statistical significance was assessed via p-values. Weighted average annual percentage change (AAPC) was computed by integrating APC values proportionally to their temporal coverage, thereby capturing heterogeneous overall trends.

Addressing the nonlinear evolution of gout burden, Joinpoint regression dynamically segments trends through data-driven inflection detection, enabling precise identification of transition points and their potential associations with external drivers. To further validate robustness, Bootstrap resampling (1,000 iterations) was implemented to estimate joinpoint occurrence probabilities, retaining only statistically significant inflection points (probability >50%). Sensitivity analyses evaluated the influence of outliers on trend directions, ensuring model reliability. All statistical procedures adhered to methodological guidelines from the National Cancer Institute’s Joinpoint software (version 4.9.1.0), maintaining analytical rigor for longitudinal disease burden assessments. Finally, the BAPC model was employed to project the incidence rate of gout in East Asia through 2050.following validated methodologies for long-term burden projections in Asian populations [13]. This approach was selected due to its capacity to address complex, high-dimensional, and sparse data structures inherent in large-scale epidemiological studies such as the GBD 2021. Departing from traditional descriptive methodologies, the BAPC framework utilizes a Poisson distribution-based formulation to dynamically integrate age, period, and cohort effects. The model incorporates temporal smoothing of these parameters through second-order random walk priors, which account for their continuous evolution over time while enhancing the precision of posterior probability estimations.

A critical innovation of the BAPC methodology lies in its implementation of the integrated nested Laplace approximation (INLA) algorithm for marginal posterior distribution approximation. This technique circumvents computational limitations associated with conventional Markov chain Monte Carlo (MCMC) approaches, including chain mixing inefficiencies and convergence challenges, while maintaining rigorous computational efficiency. The model’s flexibility in handling temporal dependencies and robustness in managing age-structured population data render it particularly advantageous for long-term disease burden forecasting.

The BAPC framework synthesizes posterior distributions via Bayesian inference. It systematically integrates sample-derived data with prior knowledge of parameter distributions. This integrative approach enables robust parameter estimation and trend extrapolation. Practical implementation was achieved using the BAPC and INLA packages within the R statistical environment. The model’s validity has been extensively demonstrated in epidemiological research, particularly in studies requiring simultaneous analysis of temporal trends, demographic structures, and generational cohort effects [14].

## Results

Based on the GBD database, there were approximately 33.57 million gout prevalent cases in Asia in 2021 (95% UI 26.55 million, 42.28 million), and the global ASPR in 2021 is 653.80 per 100,000 population (95% UI 526.1, 810.5), and the ASIR is 109.07 per 100,000 population annually (95% UI 86.38, 135.76). more information in Table 1.

**Table 1:** The number of gout cases and ASIR in Asian countries and regions in 1990 and 2021, and ASIR trends from 1990 to 2021.

Characteristics	1990		2021		1990-2021
	Incident cases (95%UI)	Incidence rate (per 100,000 population) (95% UI)	Incident cases (95%UI)	Incidence rate (per 100,000 population) (95% UI)	Percentage change in incident cases (%) rate
Global	3983109 (3178834,4911691)	93.10 (74.40,115.48)	9401585 (7438817,11731815)	109.07 (86.38,135.76)	17.16 (15.68,18.41)
High-middle SDI	987725 (787172,1220269)	95.46 (76.38,118.65)	2244859 (1774628,2821821)	120.82 (95.77,149.9)	26.56 (24.29,28.51)
High SDI	1093512 (866456,1357560)	104.6 (82.61,129.75)	2254681 (1794295,2791943)	133.71 (106.70,163.88)	27.83 (25.45,30.79)
Low-middle SDI	537185 (425090,663785)	77.00 (61.07,96.43)	1327010 (1047210,1652520)	84.05 (66.80,105.28)	9.16 (7.90,10.57)
Low SDI	202198 (159911,250554)	78.17 (62.01,97.75)	496766 (397664,616494)	82.13 (65.27,102.96)	5.06 (3.68,6.46)
Middle SDI	1159451 (923706,1430441)	95.73 (76.51,119.52)	3072463 (2428708,3860429)	110.76 (88,138.92)	15.70 (13.83,17.43)
China	1182498 (940490,1461669)	122.52 (97.99,152.96)	3079836 (2425498,3891398)	151.61 (121.16,189.2)	23.74 (21.68,25.97)
Republic of Korea	23302 (18195,29139)	127.31 (101.64,159.41)	47491 (37242,59113)	143.03 (112.62,176.84)	12.36 (6.15,18.67)
Taiwan	27227 (21887,33014)	151.39 (121.80,184.58)	66502 (52975,83803)	177.28 (142.32,218.37)	17.1 (9.12,25.80)
Cambodia	5017 (3994,6198)	92.32 (73.08,114.57)	15301 (12192,19060)	106.72 (85.29,131.94)	15.60 (8.42,22.11)
Indonesia	122798 (96606,151380)	103.35 (82.34,128.94)	350045 (275421,436794)	126.75 (101.03,159.42)	22.64 (20.19,25.29)
Lao People’s Democratic Republic	2369 (1892,2938)	98.73 (78.27,123.13)	6670 (5291,8223)	118.40 (94.47,147.09)	19.93 (14.31,26.94)
Malaysia	12445 (9980,15521)	109.20 (87.41,137.35)	41250 (32780,50841)	132.74 (105.32,163.95)	21.55 (15.32,29.08)
Maldives	121 (95,151)	112.38 (89.36,140.71)	692 (547,890)	138.45 (108.42,174.67)	23.2 (15.64,30.63)
Myanmar	26194 (20415,32449)	98.31 (77.80,122.45)	59330 (46390,73479)	111.73 (87.64,137.93)	13.65 (7.45,19.98)
Philippines	35857 (28442,44463)	98.86 (78.98,123.59)	113259 (89297,140810)	120.55 (95.58,151.27)	21.94 (20.07,23.70)
Sri Lanka	13566 (10792,17039)	107.21 (84.80,134.36)	31668 (25141,40033)	119.54 (95.58,149.81)	11.50 (5.87,18.27)
Thailand	46545 (37793,57190)	108.39 (87.11,132.71)	129901 (101340,165078)	127.91 (100.57,159.83)	18.01 (10.51,25.57)
Democratic Republic of Timor-Leste	407 (320,518)	102.14 (81.61,128.93)	1052 (830,1312)	112.98 (89.37,141.58)	10.61 (4.91,16.74)
Vietnam	41163 (33205,50659)	93.09 (74.5,116.37)	118704 (92858,148049)	109.77 (86.64,137.50)	17.92 (12.38,24.03)
Armenia	2099 (1673,2647)	71.73 (57.51,90.09)	3480 (2722,4434)	85.52 (67.77,108.60)	19.23 (12.81,26.85)
Azerbaijan	3906 (3104,4855)	71.33 (56.66,89.57)	9731 (7567,12250)	86.11 (68.23,108.48)	20.71 (15.07,29.13)
Georgia	4565 (3618,5687)	74.09 (58.65,93.31)	4331 (3462,5480)	81.91 (65.16,102.79)	10.56 (5.29,16.64)
Kazakhstan	9916 (7898,12323)	72.95 (58.29,91.41)	16276 (12774,20537)	85.16 (67.61,107.46)	16.74 (10.94,22.79)
Kyrgyzstan	2228 (1768,2803)	70.52 (56.32,88.87)	4360 (3389,5421)	79.39 (62.67,98.20)	12.57 (5.79,19.49)
Mongolia	872 (693,1082)	73.45 (58.35,92.46)	2198 (1718,2779)	79.42 (63.48,100.17)	8.13 (2.31,14.39)
Tajikistan	2049 (1630,2545)	67.96 (53.87,85.63)	5352 (4230,6732)	76.57 (60.9,95.36)	12.66 (6.46,18.44)
Turkmenistan	1582 (1262,1962)	73.34 (58.03,92.33)	3942 (3044,4949)	87.55 (68.58,109.75)	19.37 (13.15,26.51)
Uzbekistan	9303 (7449,11472)	73.36 (57.81,92.31)	25509 (20030,31607)	85.02 (67.71,105.71)	15.89 (9.69,22.62)
Brunei Darussalam	156 (125,194)	103.23 (81.85,127.91)	499 (388,631)	112.32 (88.74,140.99)	8.81 (1.77,14.62)
Japan	176965 (138855,225139)	107.32 (84.52,134.92)	292600 (228340,374291)	116.69 (91.91,147.52)	8.73 (7.4,10.05)
Republic of Korea	34248 (26593,42613)	93.99 (74.01,118.73)	90755 (70548,115114)	107.16 (84.03,133.97)	14.02 (7.75,19.44)
Singapore	2636 (2074,3303)	97.10 (76.55,121.95)	9797 (7634,12370)	115.05 (90.34,144.09)	18.48 (11.79,25.48)
Cyprus	622 (486,769)	75.50 (59.24,93.4)	1540 (1213,1920)	80.10 (63.66,99.04)	6.10 (0.19,12.55)
Israel	3668 (2918,4598)	78.72 (61.88,98.22)	9246 (7241,11702)	84.80 (66.86,106.88)	7.73 (1.69,14.03)
Bahrain	283 (221,356)	95.77 (75.75,119.39)	1624 (1256,2069)	113.52 (89.79,141.87)	18.53 (11.34,25.4)
Islamic Republic of Iran	25817 (20321,32005)	85.96 (68.12,107.38)	83979 (66223,105369)	97.11 (76.89,122.26)	12.98 (11.21,14.61)
Iraq	8214 (6554,10205)	88.95 (70.56,112.21)	28747 (22506,36118)	98.66 (77.65,124.07)	10.91 (5.12,16.86)
Jordan	1526 (1203,1886)	90.04 (70.88,112.48)	10159 (7984,12674)	109.52 (87.45,137.75)	21.63 (15.03,28.2)
Kuwait	1043 (813,1318)	105.01 (82.81,132.87)	5028 (3879,6480)	115.84 (92.27,144.4)	10.32 (3.57,16.82)
Lebanon	1920 (1501,2432)	82.55 (65.70,104.37)	5767 (4624,7159)	95.86 (76.83,119.19)	16.12 (10.64,22.65)
Palestine	780 (624,970)	79.46 (63.17,100.39)	2980 (2366,3683)	93.63 (74.28,115.96)	17.84 (11.87,23.84)
Oman	856 (674,1079)	84.06 (66.4,105.92)	3856 (2981,4964)	109.78 (86.21,136.95)	30.59 (22.7,39.57)
Qatar	297 (227,391)	110.70 (88.01,137.25)	3208 (2429,4167)	130.41 (102.73,162.7)	17.80 (10.67,24.6)
Saudi Arabia	7363 (5790,9248)	92.97 (73.69,117.6)	36532 (28254,46722)	119.29 (95.06,151.76)	28.31 (21.82,35.9)
Syrian Arab Republic	5181 (4103,6421)	85.41 (68.26,106.03)	13406 (10552,16895)	95.56 (76.3,119.8)	11.89 (5.01,17.54)
Turkey	32525 (25773,40043)	82.95 (65.18,102.36)	93211 (73077,117785)	97.20 (76.19,122.52)	17.17 (11.46,23.7)
United Arab Emirates	1112 (854,1447)	105.85 (84.78,132.56)	13866 (10073,18535)	139.39 (109.88,178.47)	31.68 (23.05,41.84)
Yemen	4209 (3339,5253)	71.01 (56.14,88.95)	14369 (11464,17776)	80.71 (63.68,101.32)	13.65 (7.18,19.28)
Afghanistan	5221 (4098,6655)	75.46 (59.96,95.17)	11078 (8636,14020)	82.99 (65.83,104.34)	9.98 (3.9,16.44)
Bangladesh	39950 (32376,49579)	73.33 (58.73,92.2)	113151 (90066,142364)	77.19 (61.94,97.39)	5.27 (−0.23,11.41)
Bhutan	221 (176,273)	72.86 (57.37,91.51)	556 (444,695)	83.25 (65.66,105.07)	14.25 (8.41,21.4)
India	414246 (328672,512665)	77.63 (61.6,97.09)	1065035 (843417,1330198)	83.23 (66.05,104.36)	7.21 (6.05,8.51)
Nepal	7720 (6060,9584)	71.23 (56.92,89.72)	19320 (15128,24145)	77.12 (60.76,96.85)	8.27 (2.94,13.56)
Pakistan	44659 (35699,55462)	71.78 (57.03,89.43)	121625 (95189,151398)	83.70 (66.03,105.14)	16.62 (12.34,21.08)

**Fig 1:**
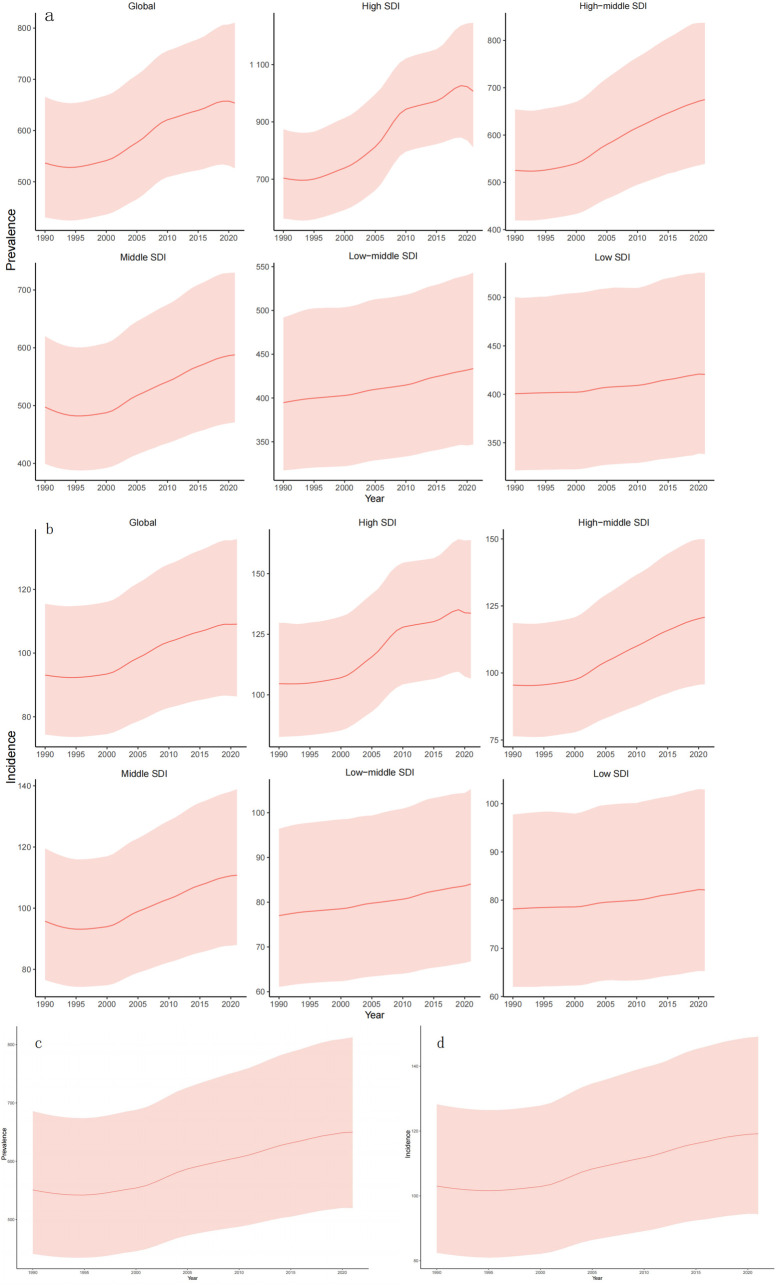
Prevalence (a), annual incidence (b) in each SDI region from 1990 to 2021, and prevalence (c), annual incidence (d) in Asian countries and regions.

**Fig 2:**
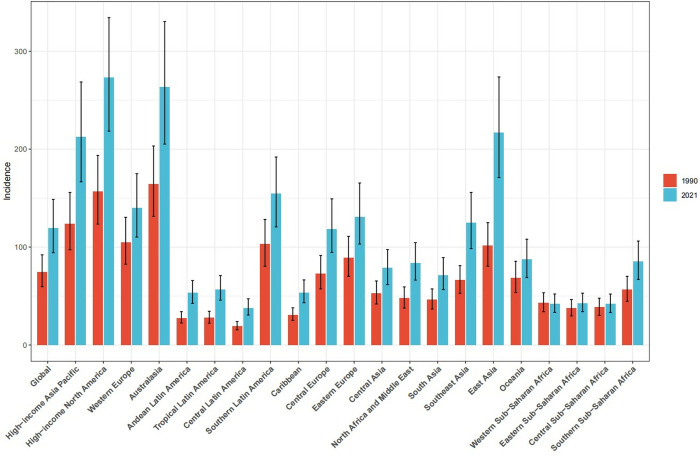
The degree of change in the global and regional annual incidence rates between 1990 and 2021.

Globally, the ASPR in 1990 was 536.54 per 100,000 population (95% UI 430.3,665.7). Compared with 1990, the ASPR in 2021 has increased by 115.28 (95% UI 95.85,144.74). ASIR was 93.10per 100,000 population annually (95% UI74.40, 115.48) in 1990 and increased by 17.16 per 100,000 population (95% UI15.68, 18.14) in 2021. The ASPR and ASIR of the world and various SDI regions are on an upward trend from 1990 to 2021. The study found that ASPR and ASIR increased rapidly in high and medium-high SDI regions, with high SDI regions experiencing the fastest growth. However, growth rates were unstable across periods: rapid development from 2000–2010, slowing from 2010–2017, and a downward trend after 2018. The rise rate of the central SDI region was slow before 2002, and the rise rate of ASPR and ASIR accelerated after 2002. Medium and low SDI areas and low SDI areas also show an upward trend, but the increase is relatively gentle. In Asia, the ASPR was 550.78 (95% UI 441.28,685.95) in 1990 and increased by 99.15 (95% UI 78.54, 126.56) in 2021. The ASIR in Asia in 1990 was 103.04 per 100,000 population annually (95% UI 82.44, 128.36), and will increase by 16.14 per 100,000 population in 2021 (95% UI 12.00, 20.86). Based on the annual incidence of gout in major regions around the world (Fig 2), the annual incidence in East Asia is growing rapidly and is much higher than the global average. The prevalence of gout in East Asia will more than double in 2021 compared to 1990. The incidence rate in South Asia is also increasing rapidly, exceeding that of most regions in the world. In addition, the DALYs in Asia in 2021 were 1,047,798 (95% UI705484, 1494747), an increase of 16.98% (95% UI15.65%, 18.38%), and the ASPR was 20.22 (95% UI 13.64,29.00). A comparison of ASPR and ASIR between the global and Asian regions found that Asia has the characteristics of late onset of disease but rapid development. Due to historically lower baseline prevalence in Asia compared to global averages, Asian prevalence rates remain lower. Notably, however, Asia’s incidence growth rate exceeds the global trend. However, it is worth noting that the growth rate of the incidence rate in Asia is higher than the global level. Incidence is growing faster, and consequently, the number of cases is also increasing relatively quickly. to control and prevent gout, the ASPR of gout in Asia may be higher than the global level in the next few years, resulting in a greater disease burden. Figs 1(a–d) and 2 illustrate these trends, particularly the accelerated growth in East Asia relative to other regions.

### Analysis based on gender and age

**Fig 3:**
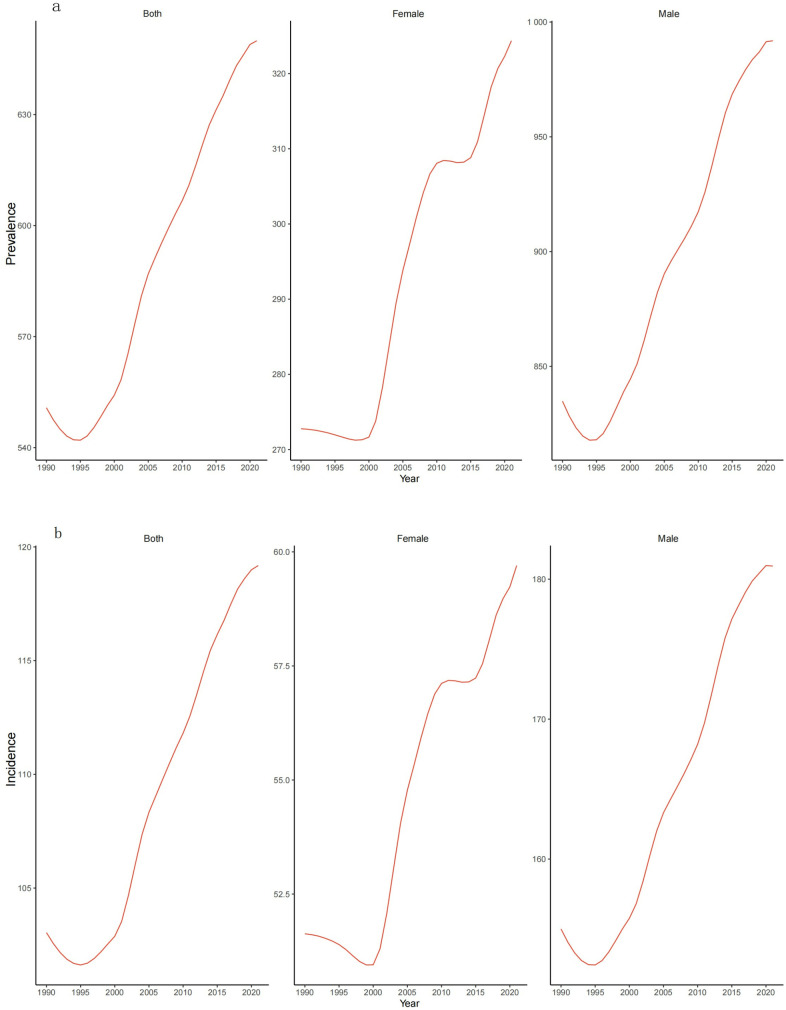
Trends in ASPR (a) and annual ASIR (b) of gout by gender in Asian regions from 1990 to 2021.

The incidence, prevalence, and DALYs of gout showed an upward trend from 1990 to 2021. In 2021, the number of male cases was 250,140 million cases (95% UI 19.80 million, 31.71 million), and the prevalence in women was 8.56 million cases (95% UI 6.76 million, 10.78 million). Men had a significantly higher prevalence compared to women The ASIR for men and women in 1990 were 155.01 cases per 100,000 people (95%UI 124.34, 193.30) and 51.63cases per 100,000 population (95% UI 41.46, 64.75) respectively. The ASIR for men and women in 2021 were 180.94 (95% UI 144.48, 277.00) and 59.70 (95% UI 47.83, 74.94) cases per 100,000 population annually, respectively. The growth rate of ASIR in males was approximately three times that in females. Men are still the main group affected by gout. In addition, the DALYs of men in 2021 are 785115.15 (95% UI 523909.26, 128723.41), and the DALYs of women are 262,68. (95% UI 176830.35, 383891.03). The DALYs of men are about three times that of women. Fig 3 can present it better. Table 2 shows the AAPC of the ASIR, ASPR and DALYs rate of gout in Asian regions and countries from 1990 to 2021 are 0.6045 and 0.6830, respectively. −0.5320 The corresponding global AAPCs are 0.5163, 0.6451, and 0.6298 respectively. This analysis indicates that both the incidence rates and prevalence of gout have increased across Asia and globally, with the growth rates in Asia exceeding the global average. In Asia, the age-standardized DALY rate showed an Average Annual Percentage Change (AAPC) of 0.5320 (95% CI: 0.4632–0.5988), lower than the global AAPC of 0.6298 (95% CI: 0.58–0.67). This suggests that while Asia’s gout burden continues to rise, its pace of increase is relatively slower than the global trend. Sex-stratified analysis revealed that the AAPC for age-standardized DALY rates among Asian males was 0.5647 (95% CI: 0.51–0.61) and 0.5429 (95% CI: 0.505–0.581) for females. Both figures were lower than their global counterparts (males: 0.6125, 95% CI: 0.57–0.65; females: 0.5949, 95% CI: 0.56–0.62). Notably, the growth rate among Asian females was 3.8% slower than Asian males, compared to a 2.8% differential globally. This regional gender disparity indicates that the progression of gout-related health burdens in Asian females lags behind the global female population. Critically, neither gender in either region demonstrated reductions in disease burden. These findings underscore the urgent need to develop targeted interventions within regional prevention strategies, incorporating both gender-specific patterns and global burden trends.

**Table 2:** Joinpoint regression analysis of the trends in the burden of gout disease in Asia and the world from 1990 to 2021.

Indicator		Asia		Global	
		AAPC	95%CI	AAPC	95%CI
ASIR	both	0.6045*	(0.55 ~ 0.65)	0.5163*	(0.48 ~ 0.54)
	male	0.6507*	(0.59 ~ 0.70)	0.4858*	(0.45 ~ 0.51)
	female	−0.1474*	(−0.19 ~ −0.09)	0.5392*	(0.46 ~ 0.61)
ASPR	both	0.6830*	(0.62 ~ 0,73)	0.6451*	(0.59 ~ 0.69)
	male	0.6701*	(0.59 ~ 0.74)	0.6206*	(0.58 ~ 0.65)
	female	0.7228*	(0.66 ~ 0.77)	0.6214*	(0.57 ~ 0.67)
DALYs rate	both	0.5320*	(0.46 ~ 0.60)	0.6298*	(0.58 ~ 0.67)
	male	0.5647*	(0.51 ~ 0.61)	0.6125*	(0.57 ~ 0.65)
	female	0.5429*	(0.51 ~ 0.58)	0.5949*	(0.56 ~ 0.62)

**Fig 4:**
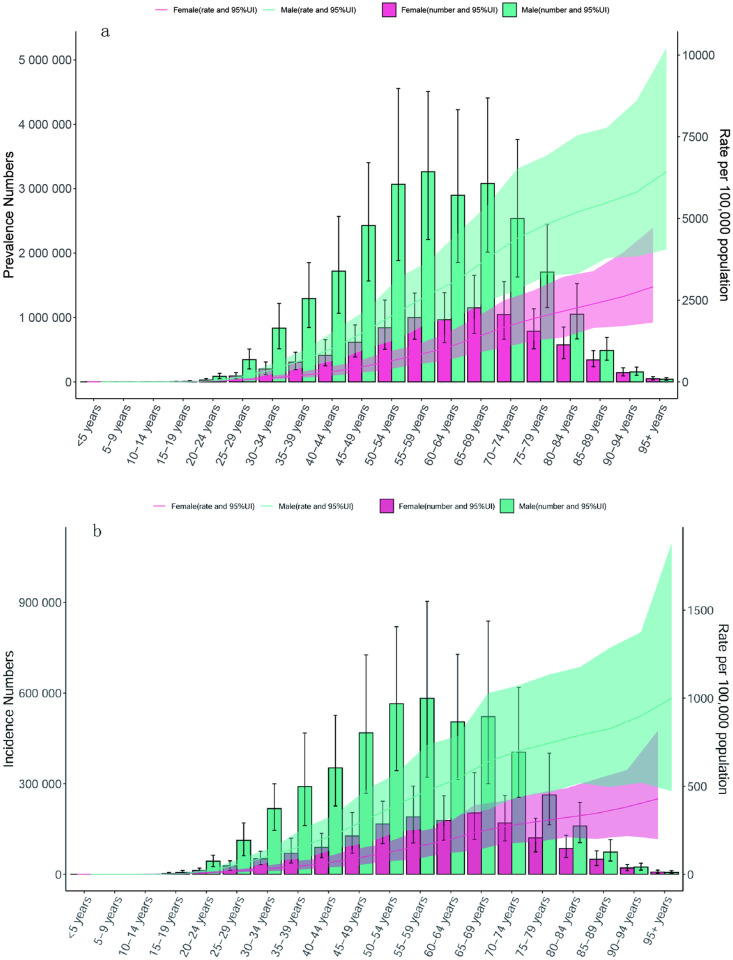
Prevalent cases and prevalence (a), and incident cases and incidence (b) of gout globally in 2021, by age and gender.

The age groups were divided into five years to study the prevalence, incidence and DALYs of gout in different genders between the ages of 15 and 84. Fig 4 shows that the prevalence among people of all ages from 1990 to 2021 is generally on the rise. Among all age groups, the prevalence is highest among those aged 55–59, with 4.26 million cases (95% UI 2.88, 5.90 million). Among them, the gap between men and women aged 50–59 is the largest. From 1990 to 2020, DALYs increased rapidly among all age groups, with the highest DALYs among those aged 55–59.

### Region-based analysis

In 2021, the prevalence, and DALYs of gout in various Asian countries and regions will generally show an upward trend. Among them, China, India, and Japan have the highest number of gout cases, with 16.79 million cases (95% UI 13.14 million, 21.28 million), 5.32 million cases (95% UI 4.22 million, 6.673 million), and 1.99 million cases (95% UI 1.57 million, 2.53 million), respectively. In 2021, the three regions and countries in Asia with the highest ASPR of gout were Taiwan, China, and Singapore, with ASPR of 1054.11 per 100,000 (95% UI 865.35, 1295.57), 810.36 per 100,000 (95% UI 644.76, 1009.05), and 805.67 per 100,000 (95% UI 634.66, 1024.80), respectively. In Asia, developed countries include Singapore, Japan, South Korea, Israel, and Cyprus. However, not all of these countries have the highest incidence rates. In 2021, the ASPR in developed Asian countries were as follows: Singapore with 805.67 per 100,000 (95% UI 634.66,1024.80), Republic of Korea with 728.59 per 100,000 (95% UI 572.95, 919.16), Japan with 722.89 per 100,000 (95% UI 568.23, 917.04), Israel with 653.19 per 100,000 (95% UI 515.37,825.34), and Cyprus with 608.07 per 100,000 (95% UI 477.36,771.84). The ASPR in Asia was approximately 650 cases per 100,000 (95% UI 520,813). Except for Cyprus, other developed countries are above the average level in Asia, but they are not ranked very high among all countries in Asia. location. At the same time, there is a certain gap between countries in terms of DALYs. Taiwan, China, and Singapore rank high. The highest age standardization of DALYs is in Taiwan, reaching 33.16 (95% UI 22.77,47.99), and the lowest is in Bangladesh at 12.27 (95%UI 17.88, 8.06), a gap of about 2.5 times.

**Fig 5:**
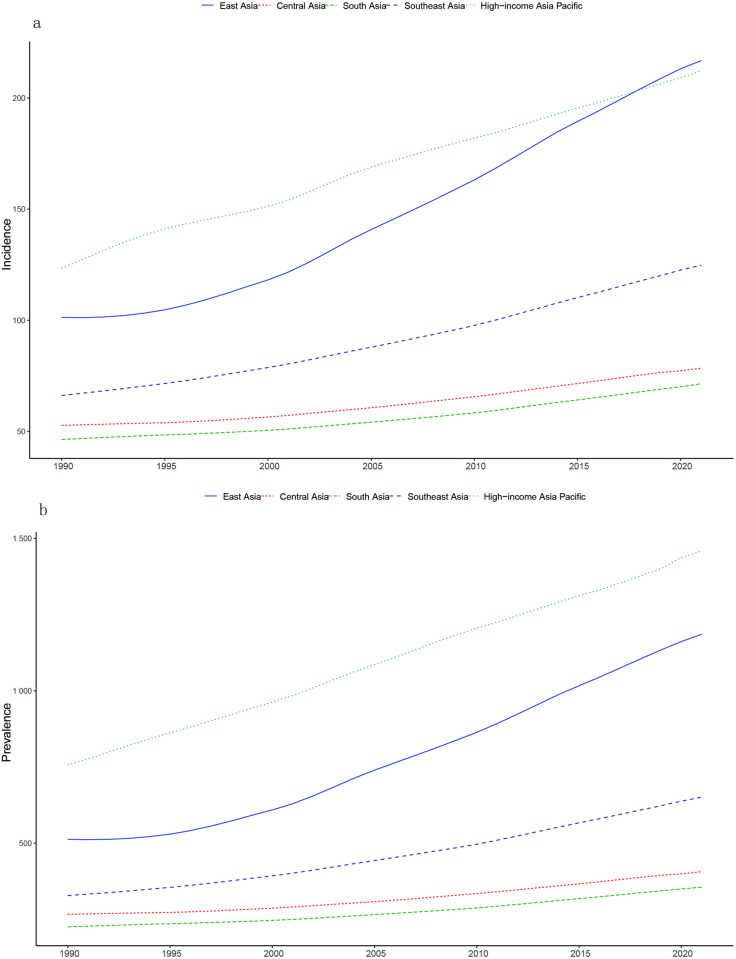
Incidence rate trends (a) and Prevalence trends (b) of gout in various regions of Asia from 1990 to 2021.

It’s easy to find that the incidence rate of gout is increasing in all regions of Asia in 2021 through Fig 5. The rate of increase is slower in Central Asia and South Asia. It is growing rapidly in East Asia and the high-income Asia-Pacific region. East Asia has the fastest growth rate and has surpassed the high-income Asia-Pacific region. Looking at the regional prevalence trends, Central Asia has seen a faster upward trend in prevalence than before, while East Asia has shown rapid growth. The prevalence in East Asia has been nearly twice that of South Asia and Central Asia. Life expectancy in the high-income Asia-Pacific region has increased significantly, which may be related to factors such as the region’s economic development level, medical technology advancements, and public health policies. Life expectancy in South Asia has increased relatively rapidly, albeit at a relatively small scale, likely reflecting the region’ s efforts to improve human health in recent years.

### Risk factor analysis

**Fig 6:**
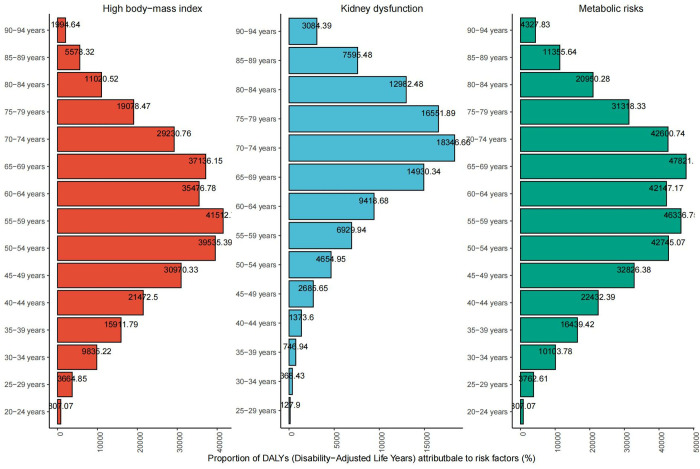
Proportional impact of risk factors for gout on DALYs by age.

Among metabolic risk factors for gout, high BMI and impaired kidney dysfunction demonstrate significant attributable risks. Among the risk factors for gout in 2021, metabolic factors account for the highest proportion, high BMI is the highest influencing factor among metabolic factors, and the proportion of impaired kidney dysfunction is relatively low. Among them, high BMI index and impaired renal function accounted for 29% (95%UI 23%, 35%) and 10% (95%UI 8%, 12%) of Years Lived with Disability (YLD). The proportions of the three are all showing an upward trend in 2021, and the proportion rankings are the same as in 1990. The proportion of metabolic causes in YLD has been increasing from 1990 to 2021, from 26% (95% UI 22%, 31%) in 1990 to 36% (95% UI 30%, 42%) in 2021. It is worth noting that compared with 1990, high BMI will grow faster in 2021. The link between gout and weight is becoming increasingly apparent. At the same time, we analyzed the impact of high BMI and impaired renal function among different genders among the risk factors leading to gout. Among all risk factors, high BMI has the largest gap between men and women. Compared with women, men have a more obvious upward trend in increased risk of gout due to high BMI. Across different regions of Asia, high BMI and impaired kidney dysfunction demonstrates similar relative importance as risk factors, although their specific attributable proportions vary. At the same time, compared with 1990, there is also a certain gap in the degree of change in 2021. In developed countries, renal impairment contributes a larger proportion to gout risk among women compared to men. Among them, the gap between the proportion of men and women in Israel due to impaired kidney dysfunction is the largest, with women accounting for 22% (95% UI 17%, 26%) and men 13% (95% UI 10%, 16%), a difference of 9% (95% 7%, 10%). Based on different age groups, we analyzed the impact of different risk factors on gout DALYs. Through the proportion and trend of risk factors, we found that the same risk factor has different effects on different age groups. The impact of each risk factor on the population has certain distribution characteristics. High BMI presents a stepwise distribution with the age group 55–59 as the axis, decreasing to high and low respectively. Impaired kidney dysfunction is greatly affected by age, and affects more concentrated groups of people. This factor has less impact on people before the age of 50, and people aged 55–84 are more affected by this factor. Among all age groups, high BMI has the greatest impact on people aged 55–59, and high BMI is also a relatively high risk factor for people aged 50–54 and 65–69. People aged 70–74 are most affected by impaired kidney function, and the impact of this factor on people aged 65–69 and 75–79 cannot be ignored. More information can be found in Fig 6.

### Predictive analysis

**Fig 7:**
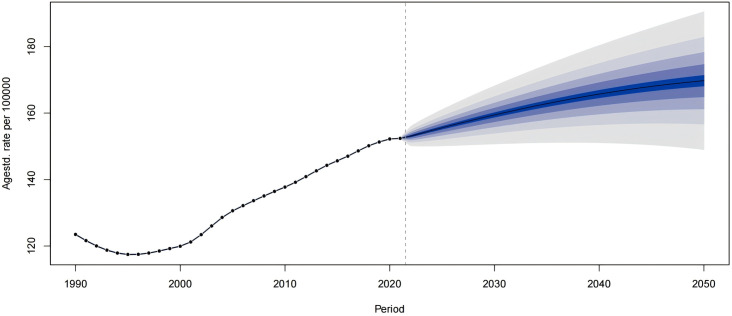
Projection of annual ASIR of gout in East Asia.

**Fig 8:**
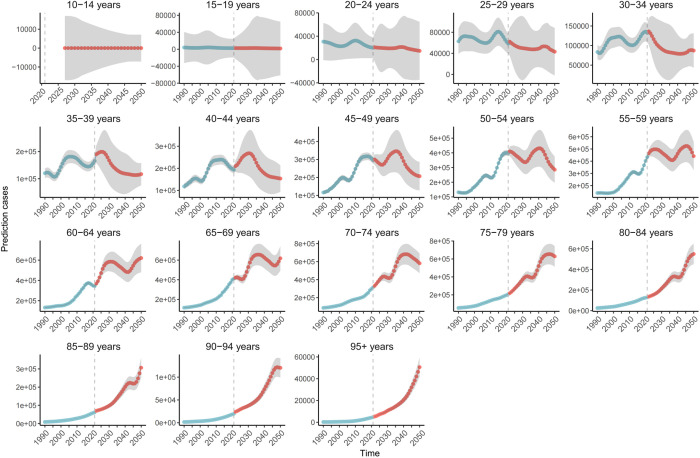
Projections of annual incidence rates of gout by age group in East Asia from 2021 to 2050.

In Asia, East Asia has the most severe burden of gout disease, with outstanding contradictions. We conducted a forecast analysis on the incidence rate of gout in East Asia (Fig 7), and found through the forecast that the annual ASIR of gout in East Asia will still show an upward trend by 2050, but the upward trend will be slower than before. At the same time, we have done predictive analysis on the East Asian population based on different age groups. The Fig 8 shows that there are differences between different age groups and different years. People aged 20–34 years old have shown a downward trend in annual incidence, those aged 35–59 years have shown a fluctuating downward trend, but people over 60 years old have shown an upward trend.

## Discussion

Gout is an inflammatory arthritis caused by disorders of uric acid metabolism, and its disease burden has increased significantly in recent years. According to data from the GBD study, the prevalence of gout and the resulting DALYs continue to increase globally. Especially in East Asia, gout patients have increased 1.76 times in the past 30 years. [15].From 1990 to 2019, the disease burden of gout in China has also increased significantly, and there are obvious gender and age differences, and gout is getting younger. It is also more obvious studies have shown that among adolescents aged 15–39, the prevalence of gout has also shown a rapid upward trend to differences in diet and geographical factors, there are regional differences in the incidence and prevalence of gout in various regions. Past research surveys on gout have focused on global prevalence, incidence, YLD values, and their correlation with 21 risk factors. There are few studies on the relationship between GBD classification regions and the disease burden of gout in Asia. Therefore, this study analyzes the incidence and prevalence of gout in Asia, and compares the incidence and prevalence of gout in Asia and the world, so as to provide an understanding of the global incidence and prevalence of gout in Asia. Understand the situation and development trends. On this basis, we conducted predictive analysis on Asian countries and regions with a severe gout disease burden to provide a theoretical basis for subsequent strategy implementation. Our research shows that the prevalence of gout in Asia showed an increasing trend from 1990 to 2021, which is consistent with the global gout incidence trend. In line with previous research conclusions. [16] Studies have found that gout is a sex-specific disease, with a higher prevalence in men than in women, and the burden of gout in men is three times higher than in women in all age groups. This disparity may be further exacerbated by comorbidities such as chronic kidney disease, which disproportionately affect men in high-risk populations [17]. In terms of age, the incidence rate is highest among those aged 55–59 years and shows a downward trend thereafter. It is consistent with previous research [2]. However, emerging evidence indicates a rising prevalence among younger adults (aged 15–39 years) in Asia, potentially linked to early-onset obesity and sedentary lifestyles [18]. In age-stratified analyses, the pronounced impact of elevated BMI on individuals aged 55–59 years may be associated with multiple biological and lifestyle factors. From a biological perspective, this age group typically experiences declining metabolic function and heightened insulin resistance, leading to increased uric acid production and altered distribution. Concurrently, age-related reductions in renal blood flow and diminished tubular function exacerbate impaired uric acid clearance, establishing a pathological cycle. Lifestyle factors further compound these risks: middle-aged populations often face substantial occupational stress coupled with decreased physical activity and increased consumption of high-calorie diets, predisposing them to central obesity. Visceral fat accumulation demonstrates a direct correlation with elevated serum uric acid levels. Furthermore, the frequent co-occurrence of metabolic disorders such as hypertension and diabetes in this demographic promotes urate crystal deposition through inflammatory responses and endothelial dysfunction. Notably, the BMI effect appears more pronounced in males within this age range, potentially attributable to androgen-mediated disparities in adipose tissue distribution. The incidence rates among people of different age groups show different trends. For young people, improving lifestyle and dietary habits may be an effective way to reduce the incidence of gout. For the elderly, in addition to lifestyle adjustments, they also need to strengthen the management and control of chronic diseases to reduce the risk of gout attacks. According to regional divisions, there are large spatial differences in the incidence and prevalence of gout. Among all countries and regions in Asia, the burden of gout disease is relatively serious in Taiwan, China, and Singapore. At the same time, the incidence and prevalence of gout in other developed countries are mostly higher than the average level, which is in line with the previous disease burden ratio in developed regions. The conclusion is that the disease burden in developing countries is more serious. At the same time, there are more developing countries among the countries and regions ranked higher. The heterogeneity in gout burden across countries may stem from the combined effects of dietary patterns, genetic susceptibility, and healthcare system capacities. In terms of diet and lifestyle, high purine intake and urbanization exacerbate metabolic risks: regions such as Taiwan and Singapore rank among the highest globally in per capita seafood consumption [16], while elevated intake of red meat and sugary beverages further enhances uric acid production through purine metabolism. In East Asian countries like Japan and South Korea, beer consumption culture accelerates uric acid synthesis due to nucleotide components in beer [2]. Concurrently, high sociodemographic index (SDI) countries such as Singapore face visceral fat accumulation caused by sedentary lifestyles, activating the NLRP3 inflammasome and triggering chronic inflammation—a critical contributor to gout pathogenesis. Genetic backgrounds also markedly drive regional disparities: the SLC2A9 gene rs734553 TT genotype prevalent in East Asian populations reduces uric acid excretion efficiency by 35%, increasing gout risk by 2.3-fold compared to other ethnic groups [19]. Meanwhile, the HLA-B*58:01 allele, frequently observed in Southeast Asian populations, is associated with severe allopurinol hypersensitivity reactions [2], limiting pharmacological options and indirectly exacerbating disease progression. Furthermore, disparities in healthcare system capabilities amplify regional imbalances: high-SDI countries (e.g., Singapore, Japan) achieve early detection and effective management through nationwide health screenings and community interventions (e.g., dietary guidance and exercise programs) whereas low-income regions like Cambodia lack basic diagnostic infrastructure and systematic chronic disease management frameworks, leading to high rates of underdiagnosis and untreated cases. The interplay of these multidimensional factors underscores the necessity for tailored, region-specific strategies in gout prevention and control across Asia. However, the accuracy of GBD data exhibits significant regional disparities. Central Asian and South Asian countries rely on model-based estimates for gout burden due to scarce primary data, leading to wide confidence intervals in ASPR. While high-SDI countries benefit from advanced surveillance systems, they may overestimate burden due to excessively sensitive diagnostics; low-SDI countries conversely underreport cases because of limited diagnostic capacity. Additionally, cultural differences complicate cross-national comparisons. At the same time, our in-depth research found that among the many risk factors that cause gout, high BMI accounts for a significant proportion, specifically as high as 29% of the total. In addition, we observed the changing trend of the proportion of high BMI among gout patients during the time span from 1990 to 2021. The results show that this proportion shows a clear upward trend, which not only reflects the increasingly severe global obesity problem, but also further confirms the increasingly close connection between high BMI and the incidence and prevalence of gout. Mendelian randomization studies have confirmed a direct causal relationship between elevated BMI and urate accumulation, independent of confounding variables [20]. In the forecast analysis, we found that by 2050, the ASIR of gout in East Asia will continue to maintain an upward trend, but the growth rate will slow down compared to the past few decades. This may be related to the increase in people’s health awareness, improvement in lifestyle and advancement in medical technology in recent years. In the next few decades, as the proportion of the elderly population increases throughout the world, the number of patients with gout, a disease that mostly affects middle-aged and elderly people, is expected to continue to rise. The elderly are more prone to reduced uric acid excretion and urate deposition due to factors such as decreased physical function and slowed metabolism, thereby increasing the risk of gout attacks. Although aging itself is one of the direct factors leading to the increase in the prevalence of gout, unhealthy lifestyles (such as high-purine diet, lack of exercise, excessive alcohol consumption, etc.) are also common in the elderly population, and these factors further aggravate the occurrence of gout. and development.

## Limitations

While GBD employs state-of-the-art statistical modeling techniques, several notable limitations should be acknowledged. This study solely relies on descriptive analysis of the GBD 2021 database, where disease burden estimates are derived from computational models rather than direct observational data. Although DALYs offer a comprehensive view of health loss, their reliance on mortality data and disability weights restricts their applicability to non-fatal conditions like gout, particularly in regions with unequal access to healthcare.

Additionally, the study only characterized high BMI and kidney dysfunction as risk factors for gout, neglecting well-established associations with lifestyle factors such as diet, alcohol consumption, and smoking. Furthermore, temporal trends may be influenced by changes in diagnostic practices over time, and some gout estimates could be underestimated due to potential underreporting. It is important to note that the upcoming 2023 dataset from the GBD may have potential implications for the findings of this research. Although this study systematically analyzed the burden of gout in Asia from 1990 to 2021 based on the GBD 2021 data, the 2023 dataset may incorporate updated demographic statistics, adjustments in disease diagnostic criteria, and regional epidemiological surveillance data, particularly new evidence regarding lifestyle changes post-pandemic and adjustments in healthcare resource accessibility. Moreover, the new dataset may provide more granular subregional stratification of disease burden data, further revealing regional heterogeneity. Although the complete data for 2023 has not yet been obtained, this potential update will contribute to a more precise depiction of the gout prevalence trends and their associations with risk factors. Future research could incorporate this dataset to further validate the predictive conclusions of this study, thereby enhancing the timeliness and specificity of gout prevention and control strategies in Asia.

## Supporting information

S1 Table Data sources used to produce Table 1 on gout incidence in Asia from 1990 to 2021. (XLSX)

S2 Table Data sources used to produce Fig 1 Global gout incidence prevalence in SDI regions and Asia, 1990–2021. (XLSX)

S3 Table Data sources used to produce the comparison chart of gout incidence in different regions between 1990 and 2021 in Fig 2. (XLSX)

S4 Table Data sources used to produce Fig 3 on the incidence and prevalence of gout by gender in Asia from 1990 to 2021. (XLSX)

S5 Table Data sources used to produce Asian and global ASIR and ASPR in Table 2. (XLSX)

S6 Table Data sources used to produce Fig 4 on the incidence and prevalence of gout in Asia by age and gender. (XLSX)

S7 Table Data sources used to produce Fig 5 Incidence and prevalence trends in different regions of Asia. (XLSX)

S8 Table The same data source used to produce Fig 6. The impact of gout risk factors in different age groups. (XLSX)

S9 Table Used as the data source to make the predictions in Fig 7. (XLSX)

S10 Table The data sources used to create Fig 8 for predicting the prevalence among different age groups in East Asia. (XLSX)
